# Emotions, action strategies and expectations of health professionals and people with dementia regarding COVID-19 in different care settings in Switzerland: a mixed methods study

**DOI:** 10.1186/s12877-023-04315-0

**Published:** 2023-10-06

**Authors:** Steffen Heinrich, Inga Weissenfels, Adelheid Zeller

**Affiliations:** Dementia Competence Centre, IPW Institute of Applied Nursing Science – Eastern Switzerland University of Applied Sciences, Rosenbergstrasse 59, St.Gallen, 9001 Switzerland

**Keywords:** Dementia care, Gerontology, Crisis intervention, Nurse-patient relationship, Nurse role, Covid-19

## Abstract

**Background:**

More than 55 million people are currently affected by dementia worldwide and over 144 thousand in Switzerland. In Swiss nursing homes, 47.6% of the residents had a medical diagnosis of dementia in 2014. Due to cognitive impairment, they have difficulties remembering hygiene measures or placing them in the epidemic context. This results in a higher infection risk. There are COVID-19-associated recommendations focused on dementia care management but studies simultaneously surveying and correlating perspectives of health professionals as well as people with dementia across care settings are largely lacking. This study is focused on COVID-19-associated perspectives and needs of health professionals and people with dementia across different care settings. Lessons learned from the pandemic shall be pointed out.

**Methods:**

We conducted a mixed-methods approach based on an exploratory sequential design. Two qualitative interview rounds (n = 15 participants) and a quantitative online survey (n = 148 participants) with people with dementia, caring relatives, Advanced Practice Nurses and nursing home managers (health professionals) were performed. Data collected was performed in nursing home and home-care settings. The SQRQ checklist was used.

**Results:**

Fear and uncertainty were highest at the beginning of the pandemic among the interviewed nursing professionals and nursing home managers. As a positive side effect of the pandemic, increased cohesion in care teams was reported. Some people with dementia experienced the decelerated outside world as pleasant and less challenging to master. Particularly during the first wave, nursing home managers rated political decision-making processes as being too slow, partly non-transparent, inconsistent, and sometimes inappropriate for people with dementia.

**Conclusions:**

Although the identified emotional and physical consequences of the COVID-19 pandemic are mostly negative for health professionals and people with dementia, research should also investigate potential positive side effects. Furthermore, political decisions should be passed on to care institutions as promptly, transparently, and comprehensibly as possible. The results provide guidance on dementia-focused COVID-19 management interventions incorporating lessons learned and considering the emotional impact of the pandemic in Switzerland and beyond.

**Supplementary Information:**

The online version contains supplementary material available at 10.1186/s12877-023-04315-0.

## Background

Older people are particularly vulnerable to the effects of a COVID-19 infection, because of often exiting multimorbidity and therefore higher risk for sever illness progression [1]. Among this group, people with dementia (PwD) require special attention. Due to cognitive impairment, affected people have difficulty remembering hygiene measures or placing them in the pandemic context so that they make sense for them [2]. Therefore, Dementia is often associated with an even higher risk of COVID-19 infection and increased mortality [3]. This dual concern of dementia and COVID-19 has raised great concerns about PwD.

According to WHO data from 2023, more than 55 million people worldwide are affected by dementia [4]. The Swiss prevalence data speak of more than 144 thousand PwD [5]. Both data reports assume that the number of unreported cases is high. Based on data from 2014, 47.6% of residents in Swiss nursing homes had a medical diagnosis of dementia and it is likely that this number has continued to rise to date due to the growing number of older people in Switzerland [6, 7].

Recurrent infections waves of COVID-19 resulted in extraordinarily high rates of infection and mortality in PwD in Switzerland and beyond [2, 8, 9]. Therefore, nursing homes were particularly affected by this crisis [7]. In Switzerland, three main waves of infection have been distinguished. The first wave began in May 2020, the second wave followed in October 2020 and the third wave in April 2021 [9]. In many cases, strict exclusion or isolation measures in nursing homes were implemented to protect the older people from infection. These measures were implemented in Switzerland during the first wave and are internationally comparable in various European and Western countries [10, 11]. This had drastic psychological and physical consequences for the residents with and without dementia and did not correspond to person-centered care [10, 12, 13]. Regarding PwD, the resulting loneliness and difficulties in practicing dementia-associated rituals (e.g., walking around) were important stress factors. This situation often intensified behavioral and psychological symptoms such as restlessness, depression or sleep disorders [14]. Therefore, nurses in long-term care facilities experienced psychological and physical strain. They were confronted with a persistently high workload as well as ethical dilemmas (e.g., self-determination vs. protection against infection of the PwD) [15–17].

For epidemic crisis situations, adapted and evidence-based recommendations for person-centered care with a focus on PwD are necessary. Not only epidemiological determinants have to be considered but also ethical aspects regarding the well-being of PwD.

In-depth knowledge of optimized care processes in nursing homes could foster individual well-being of PwD and reduce the crisis-related burden on caregivers in an epidemic situation.

Currently (April 2022), there are several recommendations for the nursing management with regard to epidemic crisis situations in the context of dementia care [16, 18]. However, studies simultaneously surveying and correlating the perspectives and needs of health professionals and PwD across different care settings are largely lacking [17].

The goal of the Covi-Dem study was to investigate the impact of the COVID-pandemic on health professionals caring for PwD, and, the impact of measures prescribed by public health authorities to protect residents in nursing homes in Switzerland. The study is focused on the perspective of different affected groups. It intends to contribute to implementing measures ensuring dignified living conditions for PwD and for nursing staff in Switzerland and internationally. Additionally, the results of this study will complement and/or confirm existing data and recommendations in this research area.

In this article, we report qualitative and quantitative data from interviews and an online survey with health professionals and PwD.

The research questions are:

1. Which emotions and (coping) strategies are described by health professionals and PwD in the context of COVID-19 prevention measures?
2. What are the expectations concerning the future course of the COVID-19 pandemic from the nurses´ point of view? Which professional demands are associated with these future-related expectations in the context of dementia care?

## Methods

### Study design

We adopted a mixed-methods approach with qualitative interview data and flanking quantitative data generated by means of cross-sectional surveys based on an exploratory sequential design [19]. This design is suited for overarching data integration and interpretation.

The reporting of this study is guided by the SRQR checklist (O’Brien et al., 2014), which is an equator checklist. We have attached the SQRQ checklist with page references as a supplementary file.

### Context of the researchers

All authors had an interest in the research topic as Covid-19 has a massive impact on society and the authors had a keen interest in analyzing parts of the impact and solutions in relation to this topic. They focused on caregivers and PwD as they work in dementia care research.

The authors are experienced in the field of dementia care research. All authors are experienced in qualitative and quantitative research methods. The first and third author conducted the interviews and analyzed them in discussion with the co-authors. To reduce possible bias, the coding and synthesis of the interview findings were discussed in several rounds in the group and finalized together. The first author has a health science background and is a physiotherapist. The second and third authors have a nursing science background and are registered nurses. The first and third authors are lectures at universities of applied sciences, the second author is a research assistant.

### Sampling and setting

In the qualitative part of the study, three PwD who were cared for at home by their relatives were interviewed at an outpatient day care center. They used this institution as a complementary service two days a week for two hours a day each. Furthermore, professional caregivers, nursing home managers, Advanced Practice Nurses (APN) from nursing homes and health department personnel at a cantonal level were interviewed. The heterogeneous selection of participants should contribute to a high diversity of opinions and content about the topic and the intended setting. We selected the participants by relying on our professional contacts and by means of the snowball principle. All persons who were asked were willing to participate in the interviews.

For the online survey, nursing homes were contacted using the address list publicly available in the cantons. A random sample of nursing home mangers and APN as well as hygiene specialists working in nursing homes during the pandemic were invited to participate in the study. After two weeks, a reminder was sent out and we also published an invitation to participate in the study in the newsletter of the Swiss Association for Nurses (Section Eastern Switzerland).

We asked nursing home directors to forward the questionnaire to an APN and/or a hygiene manager and/or another nurse in their institution. In this way, we intended to consider the perspective of persons with different nurse related functions in the nursing home setting. At the same time, these.

persons perform leadership functions in the areas of competence assigned to them. They were thus able to help shape measures for Covid-19 management.

### Data collection

The data collection took place between May 2020 and August 2021 (Table 1).

**Table 1:** Data collection times

Interview round 1	Quantitative Onlinesurvey	Interview round 2
May – July 2020	April – June 2021	March – Mai 2022

### Qualitative interviews 1

At the beginning, the interview guide had only a preliminary structure to ensure a high degree of openness concerning the thematic grid “emotions, action strategies and expectations” with reference to the COVID-19 pandemic.

After the first two interviews, the structure of the guide was refined and slightly adapted depending on the function and role of the interviewee. The following topics were addressed in all interviews: Preparation of the nursing homes for the pandemic, experiences with the measures to reduce the spread of the virus and protect the staff and residents, reaction of the residents and their relatives to the protective measures, greatest challenges during this time, support to what extent and by whom, greatest concerns and fears during the pandemic, quality and reliability of the information from the Department of Health, lessons learned, plans for the next wave of the pandemic.

Due to the different roles of the interview participants, data saturation was not achieved. The diversity of perspectives was the focus in the recruitment of the interviewees. However, there was a repetition and confirmation of certain themes regardless of the role and function of the interviewee.

We audio recorded all interviews and transcribed it verbatim. Field notes were integrated as far as they complemented the content. The interviews lasted between 40 and 80 min.

### Quantitative online survey (2021)

We reflected the results extracted from the individual and group interviews in coded content areas. This was the basis for developing a questionnaire-based online survey. By means of this primary.

quantitative data collection (with free text areas as qualitative elements) we intended to obtain a broader perspective with regard to the research questions.

We also differentiated between the first, second, and third pandemic wave and visualized these differences graphically.

The questionnaire for the online survey consisted of a total of 37 items (8 items concerning sociodemographic aspects). The main content-related topics were:

- How did the participants respond to the COVID-19 prevention measures in the long-term inpatient care setting (first, second and third wave)?
- How did the participants feel about coping with the COVID-19 pandemic in long-term inpatient care setting (first, second and third wave)?
- What kind of support and appreciation did the participants perceive during the COVID-19 pandemic in the long-term inpatient care setting (first, second and third wave)?

We developed the questionnaire by means of SoSci-Survey online survey software and disseminated it via an impersonalized link.

All authors had expertise in questionnaire development and the questionnaire was pretested internally with colleges out of the nursing home setting regarding understandability and consistence of the asked questions. The codebook can be found as supplementary file.

### Qualitative interviews 2

For a more in-depth analysis of certain topics, we conducted qualitative interviews approximately one year after the quantitative online survey. We intended to determine changes regarding future-related wishes in the context of the pandemic. For this purpose, our interview partners were nursing managers and a person from a cantonal health department of Eastern Switzerland. The interviewers were the same as in the first round.

### Data analysis

#### Qualitative data

To analyze the qualitative data, we performed qualitative content analysis according to Kuckartz [20]. This is a systematic approach to coding and categorizing qualitative data in order to identify patterns and to elaborate key content. The first step consists of organizing the material into content sections and codes. In a circular procedure, the statements of the interview partners are reduced to the essential content. Content analysis is well suited for interpreting multifaceted phenomena in nursing [21]. We used MAXQDA 2020 software for support. Based on the main topic of the interview guide, we followed a deductive procedure to create the code tree. We subdivided the main topic areas of the code tree under each of the three target groups (“professional caregivers” “family caregivers” and “PwD”).

Every author created one code, discussed it with the co-authors, and modified it, if necessary. In this way, we tried to eliminate inconsistencies in the data analysis.

### Quantitative data (questionnaire)

We analyzed the questionnaires descriptively and in parts conclusively by means of the statistical software IBM SPSS v28. The quantitative survey was an extension of the qualitative data, so the analysis focused on descriptive statistics and did not include further statistical analyze methods. To analyze survey data, we used frequencies (percentages) and median (standard deviation) for nominal and categorical variables. Additionally, we used mean and standard deviation for continuous variables. Missing values were reported for each item. The quantitative data were analysed by the second and third authors. Some of the analyses were cross-checked by both authors to validate the data analysed and the results.

### Mixed-methods analysis

**Fig. 1:**
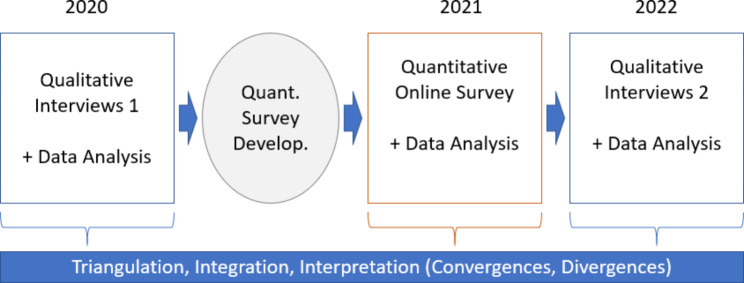
Data triangulation according to exploratory sequential design

During the exploratory sequential analysis, we first collected qualitative interview data and analyzed them to get an overview of the topic area and to find out which items can be useful for a quantitative survey. Thus, it was possible to answer the research questions on a numerically broader data basis, compared to qualitative interviews alone. After performing and analyzing the quantitative survey, we conducted further qualitative in-depth interviews. Following the Convergent Parallel Mixed-Methods Design, we identified and discussed convergences and/or divergences regarding the different qualitative and quantitative data. An exploratory sequential analysis is particularly suitable to explore unfamiliar topics over time and to develop instruments for quantitative data collection based on qualitative findings. The analysis process is displayed in Fig. 1.

## Results

### Characteristics of participants

In the context of the qualitative study, we interviewed 12 participants in the first interview round and three participants in the second interview round. The participants determined the location. A group interview with three PwD took place in a room of a day care center. The individual face-to-face interviews with the remaining participants occurred at a place of their preference. Table 2 offers sociodemographic and professional characteristics of all participants.

**Table 2:** Sociodemographic characteristics of participants (qualitative interviews)

Interview no.	Interview partner	Sex	Age (years)	Highest level of education / profession
1^†^	Relative: wife	Female	76	Teacher
2^†^	Relative: daughter	Female	59	Enrolled nurse
3	Nursing home management	Male	37	Not recorded
4	Nursing home management	Female	52	Registered nurse
5	Ward Manager Dementia Unit	Female	58	Registered nurse
6^†^	Nurse and Relative: daughter	Female	50	Master of Advanced Studies in Gerontology
7	Head of Nursing Development Cantonal Department of Health	Female	40	Master of Science in Nursing
8	Head of the Nursing Homes Division in the Department of Health	Female	59	Master of Science in Business Administration
9	Counseling dementia clarification	Female	55	Master of Advanced Studies in Gerontology
10*	PwD	One Female Two Male	58 62/63	Not recorded
11	Nursing home management	Female	44	Master of Nursing Management
12	Head of Nursing Development Cantonal Department of Health	Female	42	Master of Nursing Science

^†^Relation to PwD;

*Focus group

In the quantitative survey, 148 persons from 100 nursing homes took part. In total, we contacted 138 nursing homes, resulting in a response rate of 72.5%. Of 148 participants, 87 (58.8%) have leadership positions, 48 work as nurses, and 13 (8.8%) are APN or hygiene experts. When asked about the vaccination status, 106 participants answered “yes”, and 35 (24.8%) were not vaccinated. Reasons for non-vaccination were, for example, fear of long-term effects (n = 11), vaccine scepticism (n = 8) or having already overcome a COVID-19 infection (n = 6). Over 70% of participants (n = 106) had contact with infected residents. Further sample-related data and characteristics of nursing homes are visible in Table 3.

**Table 3:** Characteristics of participants (quantitative survey)

Study population	N (%)
Gender (n = 147)	
Female	128 (87.1)
Male	19 (12.8)
Age (n = 146)	
Under 25 years	3 (2.1)
25–30 years	10 (6.8)
31–40 years	19 (13.7)
41–50 years	35 (24.0)
51–60 years	63 (45.9)
Over 60 years	9 (7.5)
Profession (n = 148)	
Nurse	48 (32.4)
Nursing home management	87 (58.8)
APN and hygiene specialist nurses	13 (8.8)
Professional experience (n = 148)	
Under one year	8 (5.4)
1–3 years	19 (12.8)
4–6 years	25 (16.9)
7–10 years	12 (8.1)
Over 10 years	84 (56.8)
COVID-19 vaccination (n = 141)	
Yes / vaccinated	106 (75.2)
No / unvaccinated	35 (24.8)
Contact with COVID-19 positive residents (n = 146)	
Yes	106 (72.6)
No	40 (27.4)

The size of participating nursing homes in the quantitative survey varied between a maximum of 30 beds (n = 13 nursing homes) and 121 to 200 beds (n = 10 nursing homes). Most of the participating nursing homes had 31 to 60 beds (n = 40 nursing homes) at their disposal. Further data of participating nursing homes are displayed in Table 4.

**Table 4:** Characteristics of nursing homes

Characteristics	N (%)
Sponsorship of the nursing homes (n = 95)	
public	50 (52.6)
private	45 (47.4)
Number of residents (n = 98)	
1–30	13 (13.3)
31–60	40 (40.8)
61–90	26 (26.5)
91–120	9 (9.2)
121–200	10 (10.2)
Number of employees (n = 95)	
Under 25	29 (30.5)
26–50	28 (29.5)
51–80	20 (21.1)
81–110	9 (9.5)
111–200	8 (8.4)
Over 200	1 (1.1)

### Emotions perceived by professionals

#### Fear and helplessness

“Fearing the consequences of a viral infection” emerged as key finding of the qualitative data analysis in the first interview round. The professional participants mentioned “fear of infection” several times in each interview. They also expressed feelings of helplessness and being overwhelmed about the correct way of dealing with the virus. At the time of the interviews, information about the dangerousness of the virus and about major routes of transmission was scarce.

The participants reported different coping strategies for dealing with fear and helplessness. These strategies ranged from trivializing the danger to strictly avoiding an infection in the private sphere and meticulously complying with recommendations of the Federal Office of Public Health (FOPH). The fear of transmitting the virus to residents sometimes manifested itself in extremely cautious behavior:

*“We have to do everything to prevent an infection of residents. This means that the bottle of wine for my father has to be quarantined for a few days to ensure that any virus on the surface of this bottle actually dies off. Flowers or a chocolate cake or things like that – everything has to be quarantined first. We really wanted to do the best and have overshot the mark. Now we should return to professionalism and evidence” (Int6_Pos. 51)*.

The fear of spreading the virus from the outside into the institution became a great burden for caregivers and led to overcautious behaviour.

*“They are overcautious and apply abstruse rules of behaviour. One colleague goes shopping with protective goggles.“ (Int9_Pos. 101)*.

**Fig. 2:**
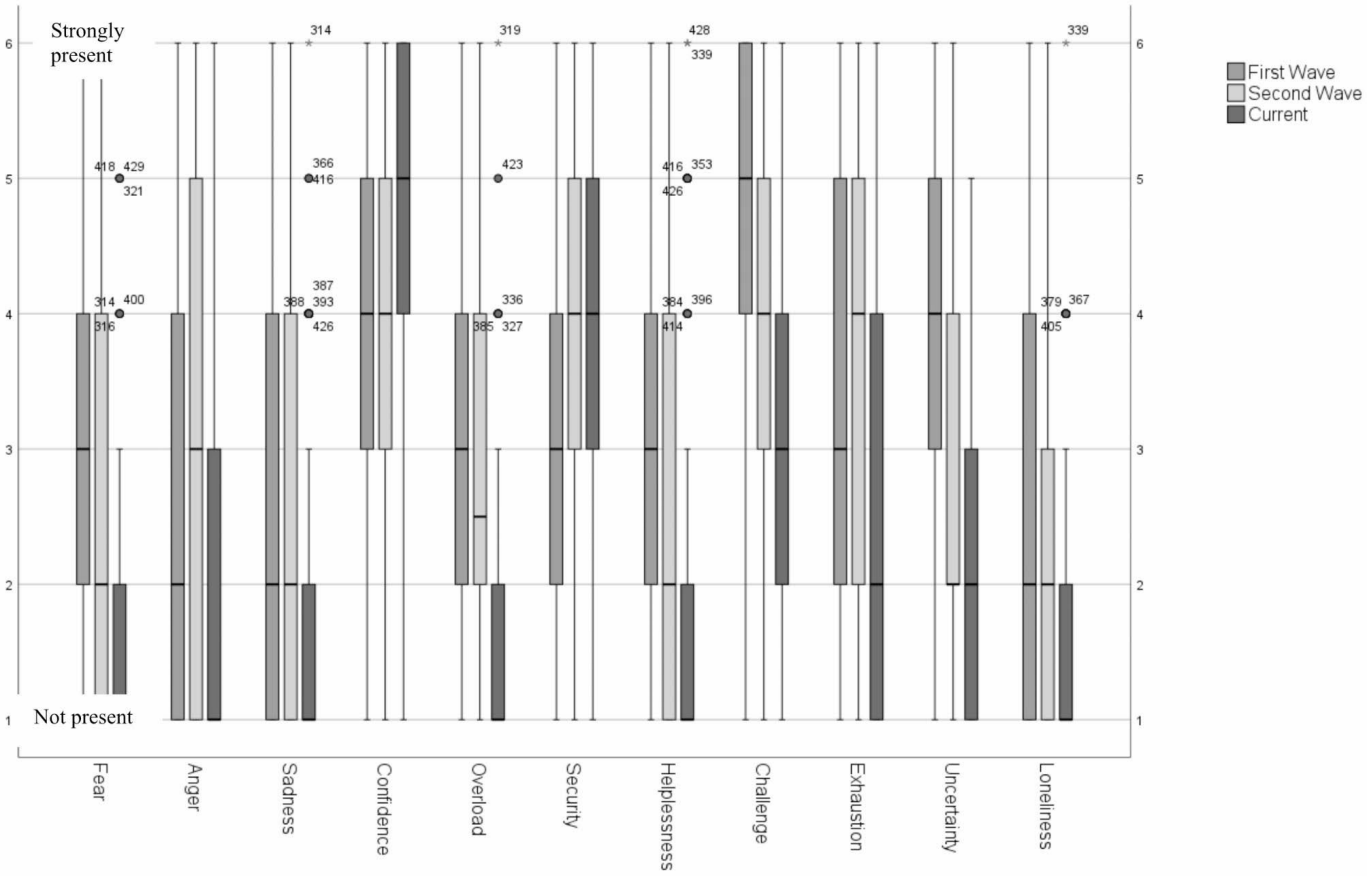
Emotions of nurses, nursing home managers and APN

In the quantitative survey, participants were asked about their emotions during the first and second waves of the pandemic as well as their current emotions. As shown in Fig. 2, around 25% felt a strong sense of fear. However, their fear significantly decreased during the second wave. Almost 23% felt very helpless during the first wave, whereas the feeling of helplessness decreased during the second wave. More than 60% said they felt very challenged during the first wave, and around 20% still feel very challenged at present. The feeling of exhaustion was particularly evident during the second wave, when Switzerland was severely affected with many people falling ill and high rates of death in nursing homes. Loneliness was felt by just under 20% of the participants during the first wave, with a decreasing tendency up to the current situation. The feeling of uncertainty was highest during the first wave with 44.1% and decreased considerably afterwards. The frequencies of further emotions are shown in Fig. 2.

Most of the participants reported a positive impact of the pandemic on teamwork and support from superiors (Table 5). In the free-text responses, however, they also noted that “initially, cooperation deteriorated” because few employees are well-trained. Therefore, they were overwhelmed, frightened and uncertain. Uncertainty also emerged due to many deaths in nursing homes. Additionally, there were few admissions during this period. This resulted in free bed capacities and worsened the financial situation of the institutions.

**Table 5:** Experience of cooperation and support by nursing staff during the COVID-19 pandemic

Support and Cooperation	N (%)
Team spirit (n = 127)	
increased	87 (68.5)
decreased	12 (9.4)
Institutional support first COVID-19 wave (n = 46)	
very satisfied	11 (23.9)
satisfied	20 (43.5)
unsatisfied	9 (19.6)
very unsatisfied	6 (13.0)
Institutional support Survey period (n = 46)	
very satisfied	13 (28.3)
satisfied	23 (50.0)
unsatisfied	7 (15.2)
very unsatisfied	3 (6.5)

Most of the participants mentioned that they felt “satisfied” or “very satisfied” with the support by their institution during the first and second infection wave. They considered the support during the second wave as even more positive.

We asked nursing home managers how they estimated the support on the part of the canton and/or the sponsorship (private or public). In this case, the perception was obviously more critical, particularly regarding the first pandemic wave. The participants mentioned that they often received information from the canton too late and that the nursing homes were simply forgotten.

**Fig. 3:**
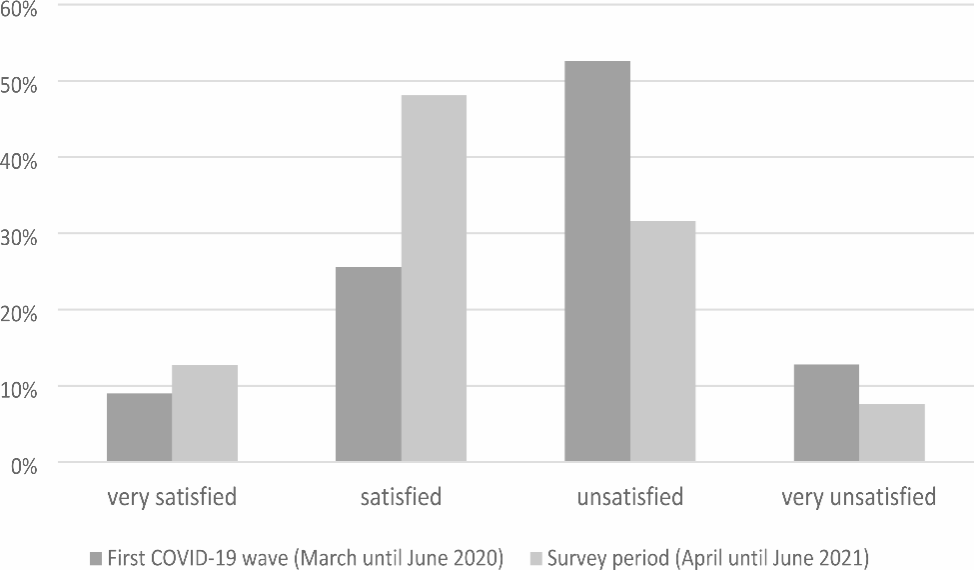
Experience of support by NH management regarding the canton and/or sponsorship

Around 65% of the respondents felt “unsatisfied” to “very unsatisfied” with the support. However, during the second wave, the support improved from the point of view of the participants (Fig. 3).

### Nurse-led COVID-19 management strategies

#### Preventing infection

During the first pandemic wave (March to June 2020) and at the time of data collection (April to June 2021) there were particularly precautionary measures in order to prevent the spread of infections.

The results clearly indicate that during the first pandemic wave, most of the participating institutions introduced contact-reducing measures (92.4%), for example a visiting ban for relatives. In addition, residents were not allowed to leave the nursing home (68.1%). Activation services were discontinued (59.7%). In case of mild symptoms, preventive quarantine was mandatory (74.3%). In 93.8% of cases, nursing homes extended their hygiene procedures. About 60% of participants reported that a crisis task force or a crisis committee was in charge. Cohorting individual living areas was another prevention measure implemented by 33.3% of the participants during the first wave (Table 6).

**Table 6:** Measures in nursing homes during the COVID-19 pandemic

Measures	First COVID-19 wave (March until June 2020) n = 144 N (% of cases)^†^	Survey period (April until June 2021) n = 130 N (% of cases)^†^
Cohorting of living areas	48 (33.3)	5 (3.8)
Freeze on admissions	25 (17.4)	0 (0)
Use of an action committee	87 (60.4)	43 (33.1)
Measures to reduce contacts	133 (92.4)	9 (6.9)
Expansion of hygienic measures	135 (93.8)	66 (50.8)
Exit ban for residents	98 (68.1)	1 (0.8)
Use of external consulting	61 (42.4)	34 (26.2)
Stop activation offers for residents	86 (59.7)	1 (0.8)
Preventive quarantine	107 (74.3)	64 (49.2)
Further Expansion of staff	17 (11.8)	45 (34.6)
Expansion / conversion of existing offerings		
Expansion of intake capacity		

^†^Multiple answers

According to the participants, preventive measures were significantly reduced during the survey period (June 2021). Thus, only 6.9% of the participants stated that contact reduction measures were still in force. Only in very few institutions (0.8%) activation services were still not allowed. In less than 50% of the institutions, preventive quarantine measures still continued. In 33.1%, a crisis task force was still in charge. None of the participating institutions had a restricted admission policy.

About half of the participating institutions introduced repetitive rapid tests to contain the transmission. The participants mentioned the following reasons for rejecting repetitive rapid tests:

- The test is performed 15 min before the beginning of a shift. In case of a positive result, the compensation of absence from work is unclear.
- Depending on the procedure, information about the result takes three to four days.
- Time-related and financial reasons.
- Divergent views among members of the management.
- Only repeated testing more than once in a week makes sense. Otherwise, it contributes to a false sense of security.

To interrupt the transmission of already occurring infections, some nursing homes segregated residents in a COVID-positive and a COVID-negative group:

*“Then the time of dying began […] Unfortunately, this was bound to happen – until we enforced the most difficult measure […]. We separated the department of dementia care into two parts – a positive one and a negative one. This was the best we could do. It resulted in enormous expenditure. There was uncertainty. The personnel was in constant fear. We got reproaches […]. But from that point onward, we interrupted the infection. We interrupted the transmission.” (Int11_Pos. 37)*.

### Resident-relative communication

Due to contact restrictions, institutions used several media to maintain communication and contact among residents. The majority used telephone (96.6%), followed by video conferencing, e.g., via Skype or Face Time (68%), e-mail (46.4%), and messenger services, such as “WhatsApp” (19.7%). Institutions may have also combined their media use since multiple responses were possible. In some nursing homes, visitors were allowed in the garden – with a fence between residents and relatives. The participants also mentioned a visitor room with a partition.

To allow visits outside the building, several facilities installed visitor pavilions. The participants mentioned separate lounges with plexiglass panes. The response was mixed:

*“At first, she didn´t want to go down to the pavilion: No, I don´t want to. Either I want to meet you properly or not at all. Then I motivated her and she was positively surprised. It´s not like a real visit in my room. I wanted to show it to her. But she made herself familiar with this”. (Int5_Pos. 58)*

### Future-related wishes of nursing staff

At the cantonal level, the minimum requirements for nursing homes are currently being revised. In this context, the required skill-grade-mix is also being addressed. Experience showed that nursing homes with an APN (Master’s degree) managed the difficult situation more effectively. It was striking that many nurses working in nursing homes were older and their training dated back more than 30 years. It is unclear how many of them attended regular further training or not.

*“In a pandemic, when suddenly expertise such as hygiene, oxygen delivery and symptom control come to the fore again, the younger ones might be a bit better trained and closer to the issues.“ (Int12_Pos. 43)*.

Nursing home managers hoped that the knowledge acquired during the pandemic will not be forgotten. It was important for them that the staff – and the population – will remain sensitized:

*“The sensitization …, the latest findings, that you really know more about droplet infection, that you remember that the mask is really the aid of paramount importance – this should really remain in the heads.” (Int11_Pos.)*

Furthermore, nursing home managers hoped that those responsible in the cantons and in the government have realized that timely decisions are decisive in critical situations:

*“It was often a burden for us that the canton, the federal government … – they always played the ball back and forth. So, I think we sometimes lost valuable time – and this resulted in uncertainties. We had somehow to decide ourselves what to do – and I think that’s always a bit of a shame.” (Int11_Pos. 53)*.

### The situation of people with/without dementia

#### Behavioural changes

The restrictions in nursing homes resulted in a significant change. The familiar daily routines of PwD changed abruptly. They experienced a reduction or even a break-off of all contacts with relatives and friends. Freedom of movement was massively restricted, particularly for PwD in nursing homes. They were not allowed to enter another ward within the institution or even to leave the institution for a walk. As a consequence, PwD exhibited increasing restlessness, wandered about the ward, and looked for their relatives. They became increasingly desperate and worried about their loved ones. One resident became convinced that his wife had died and “no one wanted to tell him the truth”. He was grieving for his wife.

Some residents had regular episodes of crying and screaming. One resident did not understand why she cannot see her son who had always supported her in difficult emotional situations. According to a nurse, this resident *“… was lost in grief and despair.”(Int6_Pos.65)*.

The abrupt change prompted PwD to stop their usual activities and give up their hobbies. This was particularly evident with regard to activities they did with their relatives. However, it affected also activities they did alone without external prompting. One daughter observed a withdrawal from usual activities with her parents. She suspected that her parents had resigned themselves to a situation seeming hopeless for them:

*“They no longer do the things they liked to do. It seems to me that they have resigned. My mother has drawing books – she paints really beautifully, three-dimensionally. She was very proud of that. Now she sits there with empty hands. How long do you want to wait?“ (Int2_Pos.20)*.

In people with very advanced dementia, the nurses observed increasingly apathetic, introverted behaviour. They suspected that this was due to masks preventing them from seeing facial expressions and responding to them:

*“He used to respond with a smile (…). But without facial expressions (because of the mask) he was very withdrawn. When he saw our faces, he would have responded – that was no longer possible now.“ (Int5_Pos.38)*.

### Declining cognitive and physical abilities

According to the participants, decreasing cognitive performance and diminished physical mobility were also observable during this time. They attributed these changes to a lack of social contact. Some of the protective measures were not feasible for residents with dementia, for example quarantine or wearing a protective mask. PwD were irritated when nurses wore masks. Indeed, some tore the masks off from the nurses´ face. As a consequence, wearing masks was not considered to be mandatory for PwD, since they would not understand why this is necessary:

*“A nursing expert [APN] told me that patients with dementia always tear off the masks from the face of the nurses (laughs). Of course, I totally understand that. Now counselling is necessary as well” (Int8_Pos. 45)*.

Mobility also declined sharply in some residents, probably due to a lack of therapies and suspended walks. Some residents consistently stayed in their rooms. Frequent nurse-led activation measures were intended to compensate for the loss of external therapeutic activation measures due to the visiting ban:

*“Yes, I think that was also discussed with the home managers and they performed in part up to 50% more activation – with members of the staff, certainly not with someone from outside. (Int7_Pos. 136)”*.

However, in most cases, nurse-led activation could not compensate for continuing physical training performed by physiotherapists and occupational therapists.

*“Their mobility has decreased a lot” (Int5_Pos. 17).*

### Emotion-related responses

PwD expressed that they are willing to accept the risk of dying. The massive restrictions on social contacts and their freedom of movement are incompatible with their need for social contact. They suffer from loneliness. Contact with their loved ones has a higher value for them than a long life:

*“Every time I speak with father on the phone, he tells me that he doesn’t want to live any more. This is simply no more life.” (Int2_Pos. 48)*.

PwD in the assisted day structure who took part in the interview after the first wave also expressed that they would rather fall ill than give up contact with their relatives or their stay in the assisted day structure.

*“So if it has to be, it has to be. I have the attitude and I am ready. I am not afraid to get sick and maybe die.“ (Int10_Pos. 174)*.

**Fig. 4:**
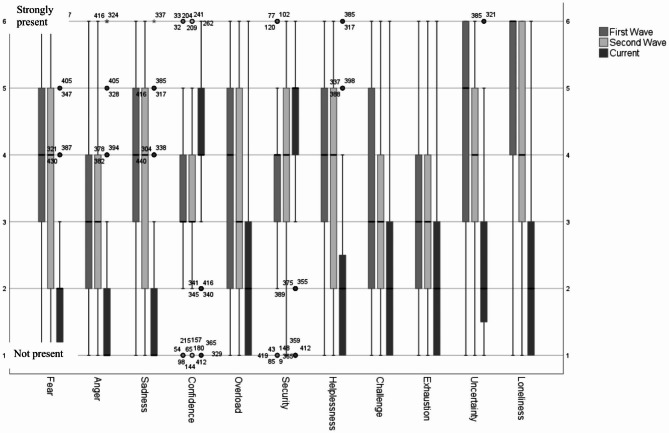
Resident’s emotions response from the nurses’ point of view

Around 70% of the participating nurses reported loneliness among the residents in the first wave and still 47.8% in the second wave. Insecurity (56.8%), helplessness (50.4%), fear (45%) and sadness (42.5) were also frequently observed emotions among residents during the first wave (Fig. 4). These and other emotions observed among residents are shown in Fig. 4.

### Safety/relaxation

Some caregivers reported that they observed relaxation in some PwD in nursing homes. They explained this by the fact that particularly people with moderate to severe dementia are confused and unsettled by visits from relatives – they can no longer recognize who the person is. This experience was largely eliminated due to visiting bans:

*“We observed that residents who had daily visits from their spouses so far were easier for us to manage. They were calmer. We care for people with moderate to severe dementia.” (Int2_Pos. 38)*.

Furthermore, some PwD perceived the calmness and less hectic pace during the lockdown as positive:

*“What I appreciated very much was the peacefulness when I was out for a walk. I really appreciated that it was so quiet. You didn’t have to be afraid of being run over. I put it so simply. You can perceive the nature.” (Int10_Pos. 12)*.

### Coping strategies

Participants also observed the development of new routines in PwD living at home with their family caregivers. For example, one people with mild dementia reported taking more extended walks to escape the confinement at home. This participant highlighted walking as very important – also to avoid potential conflicts:

*“Although we have enough space … we were too close to each other and that was exhausting for everyone. For me, it was good that I could always go for a walk. This was my rescue.” (Int10_Pos. 18)*.

For the same reason, some PwD were very happy when day care was possible again:

*“It was too much for me with my wife – and it was a relief to be able to come back to day care.“ (Int10_Pos. 41)*.

## Discussion

This study focuses on the emotional and strategic impact of the COVID-19 pandemic on nurses and PwD in Swiss care facilities. Fear and loneliness were the main emotions experienced by nurses, especially during the first waves of the pandemic, although positive emotions such as the development of a stronger team spirit and self-competence were also reported while the pandemic. PwD experienced similar feelings of anxiety and loneliness due to the new caregiving situation, although some found the slowdown and calm of the pandemic reassuring. Nursing coping strategies included infection prevention measures, increased team collaboration and increased digital communication. Cognitive decline and behavioral changes in PwD were cited as key impacts of the pandemic by nurses. Digital communication tools were experienced as negative, particularly by people with advanced dementia since they were overwhelmed by this kind of communication.

Nurses’ wishes for coping with similar crises in the future, highlighting clear political accountability, workload reduction strategies and strengthening effective care interventions were identified. Systematic maintenance and reinforcement of newly applied nurse-led measures for infection control were also desired by nursing staff. These key findings are further set in context with existing literature.

### Emotional aspects of nursing staff

#### Fear and helplessness

In the literature, fear and uncertainty have often been highlighted as an emotion that affected nurses in the context of the pandemic. A wide range of literature sources confirms this key result [22–24]. In our empirical data, COVID-19-associated fear was highest among participating nurses during the first pandemic wave. However, fear was also on a relatively low level during the first wave (25%). This could be accounted for the above-average professional training of the participants. It may be associated with higher competence in self-management.

#### Loneliness

One of our most prominent empirical findings was a frequent feeling of loneliness which resulted from partially imposed self-isolation requirements in institutions. Our findings are largely consistent with existing literature that analyzed imposed isolation processes for nursing staff in nursing homes [25]. At the same time, there are also indications in literature that intra-familial bonding processes have intensified among nursing staff in the context of the pandemic crisis [26].

#### Positive emotions

The participants of our study also mentioned positive emotional side effects of pandemic-related changes (e.g., a stronger team spirit).

Strengthened cohesion among colleagues in nursing homes as a consequence of the crisis situation and benefits from colleagues´ emotional support are also confirmed by several studies [27–29].

However, in other sources no relation between the crisis and team cohesion as a resource for nurses were discernable [30, 31].

In this respect, the overall picture proves to be inconsistent. This may be associated with the fact that team constellations and hierarchy levels are very individual constructs. Possibly, previous staff constellations have counteracted cohesion among nurses. Under conditions of increased workload these constellations may have acted even more as stressors. To confirm or refute such theses, future research is required.

### Emotional aspects of PwD from the nurses´ point of view

#### Fear and loneliness

Anxiety and loneliness were named several times by caregivers with reference to PwD in nursing homes. In the literature, fear of loneliness as a result of isolation measures and contact reduction among PwD are widely documented [29, 32].

#### Safety/relaxation

Some PwD in the homecare setting experienced the pandemic-related tranquility and deceleration of their surrounding as relaxing and soothing. Furthermore, visitation bans in nursing homes resulted in a reduction of agitated behavior of some severely dementia affected people. The identified literature almost exclusively mentions negative effects of the pandemic on PwD [13, 29]. Only one study found a reduction of depressive symptoms in a proportion of PwD [33].

### Nurse-led COVID-19 management strategies

#### Preventing infection

Particularly at the beginning, isolation measures for residents were very strict. In the literature, this phenomenon is confirmed with reference to Switzerland as well an internationally [34].

However, there was a tendency to critically reflect visitation bans and isolation measures and to avoid them as much as possible during the pandemic worldwide [34, 35]. This avoidance of isolation measures could have contributed to the massively increased mortality rates in nursing homes during the second wave in Switzerland and beyond [9, 10, 36].

#### Team cooperation and support

Collaboration and a sense of team support became stronger in most of the care teams during the pandemic in nursing homes. In a qualitative study, Hendricksen et al. noted that the staff “pulled together” to manage the crisis situation – despite staff shortages [28].

### Resident-relative communication

The use of digital communication media, such as video telephony, highly increased during the pandemic. This was an international phenomenon as shown in the literature [32, 37]. However, digital communication tools were mostly experienced as negative, particularly by people with advanced dementia, since they were overwhelmed by this kind of communication [28].

Innovative, nondigital approaches, such as visitor pavilions with separated seating areas were also common across countries in various institutions [18].

### The situation of people with/without dementia

#### Cognitive decline

On several occasions in all interview rounds, caregivers and facility managers reported that they observed cognitive decline in PwD. The decline was characterized by severe confusion and disorientation. Similar observations by caregivers are described in the literature [17, 38, 39].

#### Coping strategies of PwD

Increased withdrawal into one’s own world has been mentioned several times by caregivers of PwD in nursing homes. There are inconsistent findings in the literature. Increased apathy, depressive moods due to isolation and lack of contact were the most frequently cited behavioral phenomena during COVID-19 pandemic [32]. Interestingly, in a Swiss case study, no pronounced negative psychological effects were detected by the nursing staff regarding PwD in the hospital setting [40].

In the literature we reviewed, positively connoted action strategies of PwD in the home care setting, for example taking more walks outside the building, as mentioned in the interviews, were not described.

#### Challenging behavior

In the qualitative interview data and in the online survey data, participants highlighted behavioral changes in about one third of PwD as consequences of COVID-19-associated isolation, visiting bans and lack of exercise. Agitated behavior, disorientation, and depressive behavior were increasingly observed. This is in line with the literature. [17] and [38] described depression but also aggressive behavior by residents as COVID-19-associated behavioral changes.

#### Reduced activation measurements

The results of our study indicate that some nurses tried to compensate some of the occupational and physical therapy sessions cancelled due to the pandemic. On the other hand, however, the results of the online survey show that residents´ physical abilities were deteriorating. This was the case for residents with and without dementia. These results coincide with the existing literature emphasizing once more the importance of physical training in the form of systematic activation to maintain residents´ (as well as seniors´) functional abilities in everyday life [17, 29, 39].

### Future-related wishes of nursing staff

#### Clear responsibilities and faster political decision-making processes

With regard to nurses´ future-related wishes and demands, it became evident that clear responsibilities are desired on the political level. Furthermore, the participants also criticized delayed information due to redundant feedback loops between the canton and the federal government. Similar criticism is described for other countries [18].

#### Measures against high workload

The increased workload caused by the pandemic and inadequate measures to reduce the workload were a repeated issue in the interviews with nursing staff. The perceived insufficient response to this problem was also described as a central factor for feeling insufficiently appreciated. This kind of perception is confirmed by the literature [23].

#### Maintenance and reinforcement of newly applied nurse-led measures

Systematic maintenance and reinforcement of newly applied nurse-led measures for infection control were also desired. This demand is in accordance with other research findings. In one study, participants reported uncertainty due to constant changes in hygiene regulations. They called for a nursing home guideline specifically addressing this issue [17]. The demand for more widespread implementation of evidence-based care models is also in line with the findings of Vellani et al. [25].

#### Limitations

Our study has several limitations. In the qualitative interviews, the experiences of people in need of care (with and without dementia) were reported for the most part by means of third-party assessment by nursing staff and relatives. This was due to restricted access to people in need of care at the time of data collection in the first and second half of 2020. However, to a limited extent, it was possible to have contact with PwD in day care centers, who were cared at home the other time by their relatives and to ask them about their view on the pandemic situation. This was particularly important for us since it was our intention to give this group of people their own voice. Due to resource constraints, the last round of interviews was very limited (three participants). Nevertheless, the interview generated some important indications, particularly about future-related wishes of nursing staff.

The results of this study make no claim to generalizability. It is very likely that the comparison between the first, second and third wave is influenced by a response and recall bias, as we asked all questions during the third wave. This was one of the reasons why we only evaluated the quantitative results descriptively and not analytically. Nevertheless, it was possible to identify valuable trends regarding changes in feelings, perceptions and action strategies of the surveyed group during the pandemic.

Furthermore, we would like to emphasize once again that we only interviewed nurses and health professionals with above-average qualifications, some of them in management positions. Therefore, we can assume that the findings are only to limited extent transferable to the entire nursing staff. However, one of the aims of the Covi-Dem study was to learn more about strategies for managing the pandemic since APN and managers are an important source of information. They belong to the group of people initiating and co-implementing new measures.

## Conclusion

### Practical implications

This study emphasized the importance of efficient communication and coordination between federal and cantonal levels in Switzerland for the smooth functioning of care institutions. Timely, transparent, and comprehensible communication of political decisions can reduce uncertainty among nursing staff and enable person-centered care for care-dependent individuals. Beyond the Swiss context, the study also indicates the need for strategic measures in health care, particularly focusing on PwD in nursing homes, as “after the crisis is before the crisis.“ The insights provide directions for developing COVID-19 management measures in nursing homes, considering both nurses’ and residents’ emotional states. This balance will help in assessing whether a protective measure, such as infection control versus isolation, has too many negative consequences to be beneficial. As also pointed out by some interviewed nurses, more evidence-based interventions and processes could have helped them for the Covid management. Evidence based practice (EBP) refers to the integration of best available scientific evidence, clinical expertise and patient values into the care process. In this way, evidence-based and person-centred strategies could have been researched and implemented earlier.

Uncertainties among caregivers and PwD could have been reduced. However, EBP requires resources (e.g., for evidence research) that are often not available in everyday professional life. This is a major problem in Switzerland as well as in many other countries. Without appropriate working conditions, it will hardly be possible to provide the best possible care.

### Research implications

The study adds to the existing body of knowledge on the effects of the COVID-19 pandemic on PwD and nursing staff, with a geographical focus on Eastern Switzerland. However, the results also have validity beyond this region and the German-speaking area. The literature predominantly centers on the negative emotional and physical impacts of the pandemic. However, this study suggests the necessity of exploring aspects that facilitate positive changes. Examples include the deceleration of everyday life, which some PwD found pleasant, and the sense of intensified teamwork among nursing staff. Post the acute COVID-19 crisis, these positive factors could possibly be integrated into nursing care to contribute to person-centered practice. Additionally, the potential positive side effects of the crisis highlighted in this study, like systematic team-building measures for nursing staff and needs-oriented retreat areas for PwD, deserve independent scientific reflection and research beyond the COVID-19 related context.

However, on a global view, the results of this and similar articles showed a difficult search of the nursing staff for a balance between maximum protection against infection and the basic human needs of the residents and themselves. This has literally been a matter of life and death, as evidenced by the high death rates in nursing homes during the second wave in Switzerland and beyond. Systematic support from politics, society and science in such decision-making and crisis management processes is indispensable. It is essential that the experience gained with Covid-19 in the nursing setting and beyond gets further exchanged and reflected upon internationally.

### Electronic supplementary material

Below is the link to the electronic supplementary material.

Supplementary Material 1

Supplementary Material 2

## Data Availability

The datasets used and/or analysed during the current study are available from the corresponding author on reasonable request.

## References

[1] Becerra-Muñoz VM, Núñez-Gil IJ, Eid CM, García Aguado M, Romero R, Huang J (2021). Clinical profile and predictors of in-hospital mortality among older patients hospitalised for COVID-19. Age Ageing.

[2] Wang H, Li T, Barbarino P, Gauthier S, Brodaty H, Molinuevo JL (2020). Dementia care during COVID-19. The Lancet.

[3] Batty GD, Deary IJ, Luciano M, Altschul DM, Kivimäki M, Gale CR (2020). Psychosocial factors and hospitalisations for COVID-19: prospective cohort study based on a community sample. Brain Behav Immun.

[4] WHO. Dementia - Fact Sheet. 2023. https://www.who.int/news-room/fact-sheets/detail/dementia.

[5] BÜRO FÜR ARBEITS- UND SOZIALPOLITISCHE STUDIEN. Indikatoren «Versorgungsmonitoring Demenz» - Prävalenzschätzungen zu Demenzerkrankungen in der Schweiz. Bern; 2021.

[6] Alzheimer Schweiz. Menschen mit Demenz in Schweizer Pflegeheimen: Vielfältige Herausforderungen. 2014. https://www.alzheimer-schweiz.ch/fileadmin/dam/Alzheimer_Schweiz/Dokumente/Publikationen-Produkte/200_2014_demenz-pflegeheimen.pdf. Accessed 7 Jan 2021.

[7] Ackermann S, Baumann Hölzle R, Biller Andorno N, Krones T, Meier-Allmendinger D, Monteverde S (2020). Pandemie: Lebensschutz und Lebensqualität in der Langzeitpflege. Schweiz Ärzteztg.

[8] Bianchetti A, Rozzini R, Guerini F, Boffelli S, Ranieri P, Minelli G (2020). Clinical presentation of COVID19 in Dementia Patients. J Nutr Health Aging.

[9] WHO. WHO Coronavirus (COVID-19) Dashboard - International. 2023. https://covid19.who.int/table.

[10] Burns A, Lobo A, Olde Rikkert M, Robert P, Sartorius N, Semrau M, Stoppe G (2021). COVID-19 and dementia: experience from six european countries. Int J Geriatr Psychiatry.

[11] Sims S, Harris R, Hussein S, Rafferty AM, Desai A, Palmer S (2022). Social distancing and isolation strategies to prevent and control the transmission of COVID-19 and other Infectious Diseases in Care Homes for older people: an International Review. Int J Environ Res Public Health.

[12] Numbers K, Brodaty H (2021). The effects of the COVID-19 pandemic on people with dementia. Nat Rev Neurol.

[13] Verbiest MEA, Stoop A, Scheffelaar A, Janssen MM, van Boekel LC, Luijkx KG (2022). Health impact of the first and second wave of COVID-19 and related restrictive measures among nursing home residents: a scoping review. BMC Health Serv Res.

[14] Brown EE, Kumar S, Rajji TK, Pollock BG, Mulsant BH (2020). Anticipating and mitigating the impact of the COVID-19 pandemic on Alzheimer’s Disease and related dementias. Am J Geriatr Psychiatry.

[15] Bussè C, Barnini T, Zucca M, Rainero I, Mozzetta S, Zangrossi A, Cagnin A (2022). Depression, anxiety and sleep alterations in caregivers of persons with Dementia after 1-Year of COVID-19 pandemic. Front Psychiatry.

[16] Hoel V, Seibert K, Domhoff D, Preuß B, Heinze F, Rothgang H, Wolf-Ostermann K (2022). Social Health among german nursing home residents with dementia during the COVID-19 pandemic, and the role of technology to promote Social Participation. Int J Environ Res Public Health.

[17] Benzinger P, Kuru S, Keilhauer A, Hoch J, Prestel P, Bauer JM, Wahl HW (2021). Psychosoziale Auswirkungen der Pandemie auf Pflegekräfte und Bewohner von Pflegeheimen sowie deren Angehörige – ein systematisches review. Z für Gerontologie und Geriatrie.

[18] Ryoo N, Pyun JM, Baek MJ, Suh J, Kang MJ, Wang MJ (2020). Coping with dementia in the Middle of the COVID-19 pandemic. J Korean Med Sci.

[19] Creswell JW, Plano Clark VL (2018). Designing and conducting mixed methods research.

[20] Kuckartz U (2014). Mixed methods: Methodologie, Forschungsdesigns und Analyseverfahren.

[21] Vaismoradi M, Turunen H, Bondas T (2013). Content analysis and thematic analysis: implications for conducting a qualitative descriptive study. Nurs Health Sci.

[22] Scerri A, Borg Xuereb C, Scerri C (2022). Nurses’ experiences of caring for long-term care residents with Dementia during the COVID-19 pandemic. Gerontol Geriatr Med.

[23] Altintas E, Boudoukha A-H, Karaca Y, Lizio A, Luyat M, Gallouj K, El Haj M. Fear of COVID-19, emotional exhaustion, and care quality experience in nursing home staff during the COVID-19 pandemic. Arch Gerontol Geriatr. 2022;1–6. 10.1016/j.archger.2022.104745. 10.1016/j.archger.2022.104745 PMC9169422 35714475

[24] Husky MM, Villeneuve R, Tabue Teguo M, Alonso J, Bruffaerts R, Swendsen J, Amieva H (2022). Nursing home workers’ Mental Health during the COVID-19 pandemic in France. J Am Med Dir Assoc.

[25] Vellani S, Zuniga F, Spilsbury K, Backman A, Kusmaul N, Scales K (2022). Who’s in the House? Staffing in Long-Term Care Homes before and during COVID-19 pandemic. Gerontol Geriatr Med.

[26] Hoedl M, Thonhofer N, Schoberer D (2022). COVID-19 pandemic: burdens on and consequences for nursing home staff. J Adv Nurs.

[27] Rony MKK, Islam K, Alamgir HM (2022). Coping strategies that motivated frontline nurses while caring for the COVID-19 patients during the pandemic: a scoping review. J Nurs Manag.

[28] Hendricksen M, Mitchell SL, Palan Lopez R, Roach A, Hendrix Rogers A, Akunor H, McCarthy EP. ADVANCE-C: a qualitative study of Experiences caring for nursing home residents with Advanced Dementia during the COVID-19 pandemic. J Gerontol B Psychol Sci Soc Sci. 2022;gbac093. 10.1093/geronb/gbac093. 10.1093/geronb/gbac093 PMC9278215 35803591

[29] Giebel C, Lion KM, Lorenz-Dant K, Suárez-González A, Talbot C, Wharton E, et al. The early impacts of COVID-19 on people living with dementia: part I of a mixed-methods systematic review. Aging Ment Health. 2022;1–14. 10.1080/13607863.2022.2084509. 10.1080/13607863.2022.2084509 PMC11996062 35763444

[30] Mediavilla R, Monistrol-Mula A, McGreevy KR, Felez-Nobrega M, Delaire A, Nicaise P (2022). Mental health problems and needs of frontline healthcare workers during the COVID-19 pandemic in Spain: a qualitative analysis. Front Public Health.

[31] Dürr L, Forster A, Bartsch CE, Koob C (2021). Anforderungen, Ressourcen und Arbeitsengagement Pflegender während der zweiten Welle der COVID-19-Pandemie. Pflege.

[32] Simonetti A, Pais C, Jones M, Cipriani MC, Janiri D, Monti L et al. Neuropsychiatric symptoms in Elderly with Dementia during COVID-19 pandemic: definition, treatment, and future directions. Front Psychiatry. 2020;11. 10.3389/fpsyt.2020.579842 PMC7550649 33132939

[33] El Haj M, Altintas E, Chapelet G, Kapogiannis D, Gallouj K (2020). High depression and anxiety in people with Alzheimer’s disease living in retirement homes during the covid-19 crisis. Psychiatry Res.

[34] Dykgraaf SH, Matenge S, Desborough J, Sturgiss E, Dut G, Roberts L (2021). Protecting nursing Homes and Long-Term Care Facilities from COVID-19: a Rapid Review of International evidence. J Am Med Dir Assoc.

[35] Frazer K, Mitchell L, Stokes D, Lacey E, Crowley E, Kelleher CC (2021). A rapid systematic review of measures to protect older people in long-term care facilities from COVID-19. BMJ Open.

[36] BAG. Bericht Todesfälle im Zusammenhang mit Covid-Covid 19 in der Schweiz und im internationalen Vergleich. Bundesamt für Gesundheit; 2021.

[37] Noone C, McSharry J, Smalle M, Burns A, Dwan K, Devane D, Morrissey EC (2020). Video calls for reducing social isolation and loneliness in older people: a rapid review. Cochrane Database of Systematic Reviews.

[38] Soysal P, Smith L, Trott M, Alexopoulos P, Barbagallo M, Tan SG, et al. The Effects of COVID-19 lockdown on neuropsychiatric symptoms in patients with dementia or mild cognitive impairment: a systematic review and meta-analysis. Psychogeriatrics. 2022;402–12. 10.1111/psyg.12810. 10.1111/psyg.12810 PMC9115368 35128762

[39] Suárez-González A, Rajagopalan J, Livingston G, Alladi S (2021). The effect of COVID-19 isolation measures on the cognition and mental health of people living with dementia: a rapid systematic review of one year of quantitative evidence. EClinicalMedicine.

[40] Neumann E, Ballmer L, Studhalter O, Schmid N, Jung HH (2023). Dementia during the COVID-19 pandemic: Experiences from Clinicians, Patients, and caregivers in Switzerland. Gerontol Geriatr Med.

