# Supplementary material for: Emotions, action strategies and expectations of health professionals and people with dementia regarding COVID-19 in different care settings in Switzerland: a mixed methods study
Source: BMC Geriatr. 2023 Oct 6;23:631. doi: 10.1186/s12877-023-04315-0 (PMC10559654; doi:10.1186/s12877-023-04315-0)
Supplement: Supplementary file 1 — Supplementary Material 1 [file 12877_2023_4315_MOESM1_ESM.pdf]

DeepL
Subscribe to DeepL Pro to edit this document.
Visit www.DeepL.com/pro for more information.

Variable	Labeling	Question	Input	Measurement level	Missing values	Filter-variable
CASE	Interview number (consecutive)			Scale		
SERIAL	Serial number (if used)			Nominal		
REF	Reference (if specified in the link)			Nominal		
QUESTNNR	Questionnaire used in the interview			Nominal		
MODE	Interview mode			Nominal		
STARTED	Time at which the interview started (Europe/Berlin)			Scale		

Variable	Labeling	Question	Input	Measurement level	Missing values	Filter-variable
SD03	Age (categories, 10 years)	How old are you?	-9[a] not answered<br>-1[a] No specification<br>1 under 25 years<br>2 25 - 30 years<br>3 31 - 40 years<br>4 41 - 50 years<br>5 51 - 60 years<br>6 over 60 years	Scale	-1, -8, -9	
SD04	Gender	What gender are you?	-9[a] not answered<br>-1[a] No specification<br>1 female<br>2 male<br>3 diverse	Scale	-1, -8, -9	
SD05	Work experience	How many years of professional experience do you have in the current position?	-9[a] not answered<br>-1[a] No specification<br>1 under 1 year<br>2 1 - 3 years<br>3 4 - 6 years<br>4 7 - 10 years<br>5 over 10 years	Nominal	-1, -8, -9	
SD06	Current occupation	In which professional function are you currently working?	-9[a] not answered<br>1 qualified nurse<br>2 Division Management Nursing and Care / Management<br>3 Nursing expert / hygiene officer	Nominal	-8, -9	

Variable	*Labeling*	*Question*	*Input*	*Measurement level*	*Missing values*	*Filter-variable*
SD07	Size of the home - MA	How many employees <u>in nursing</u> does your home employ <u>(full-time positions)</u> ?	-9[a] not answered<br>-1[a] No specification<br>1 to 25<br>2 26 - 50<br>3 51 - 80<br>4 81 - 110<br>5 111 - 200<br>6 over 200	Scale	-1, -8, -9	SD06=2&3
SD08	Size of the home - BW	How many residents live in your home	-9[a] not answered<br>-1[a] No specification<br>1 to 30<br>2 31 - 60<br>3 61 - 90<br>4 91 - 120<br>5 121 - 200<br>6 over 200	Scale	-1, -8, -9	SD06=2&3
SD09	Sponsorship of the home	What is the ownership of your retirement or nursing home?	-9[a] not answered<br>-1[a] No specification<br>1 public<br>2 private	Scale	-1, -8, -9	SD06=2&3
SD10	Size of the station - BW	How many residents are on your ward?	-9[a] not answered<br>-1[a] No specification<br>1 to 15<br>2 16 - 20<br>3 21 - 25<br>4 over 25	Scale	-1, -8, -9	SD06=1

Variable	Labeling	Question	Input	Measurement level	Missing values	Filter-variable
AF01	Own vaccination status	Have you already been vaccinated?	-9[a] not answered<br>-1[a] No specification<br>1 Yes<br>2 No	Scale	-1, -8, -9	
AF02	Filter 1: Reason no vacc.	Could you tell us the reason why you don't get vaccinated?	-9[a] not answered<br>-1[a] No specification<br>1 Fear of complications after vaccination (e.g. allergic reactions).<br>2 Fear of late effects of vaccination<br>3 I still need some time to think<br>4 I already had a Covid-19 infection<br>5 Further or explanation:	Scale	-1, -8, -9	AF01≠1
AF02_05	Filter 1: Reason no vaccination: Further or explanation			Nominal		
AF03	Estimated vaccination rate MA	How high do you estimate the percentage of vaccinated employees?	-9[a] not answered<br>-1[a] No specification<br>1 To 20%<br>2 21 - 40%<br>3 41 - 60%<br>4 61 - 80%<br>5 over 81%	Scale	-1, -8, -9	
AF04	COVID - own Erkr.	Have you personally had a Covid-19 infection?	-9[a] not answered<br>-1[a] No specification<br>1 Yes<br>2 No	Scale	-1, -8, -9	
AF05	COVID Contact - Residents	Do/have you had direct contact with residents suffering from Covid-19 during your daily work?	-9[a] not answered<br>-1[a] No specification<br>1 Yes<br>2 No	Scale	-1, -8, -9	
AF06	Estimated infection sq. Bew.	What percentage of residents tested positive for Corona across the institution between April 2020 and April 2021 (estimate)?	-9[a] not answered<br>-1[a] No specification<br>1 To 20%<br>2 21 - 40%<br>3 41 - 60%	Scale	-1, -8, -9	SD06=2

			4 61 - 80%<br>5 over 81%			
AF07	Estimated vaccination rate. Bew.	What percentage of residents are now fully vaccinated in your institution (estimate)?	-9[a] not answered<br>-1[a] No specification<br>1 To 20%<br>2 21 - 40%<br>3 41 - 60%<br>4 61 - 80%<br>5 over 81%	Scale	-1, -8, -9	SD06=2
AF08	Reason no vacc. - Inhabit.	Can you tell us why residents did not get vaccinated?	-9[a] not answered<br>-1[a] No specification<br>1 Residents' fear of vaccination complications (e.g. allergic reactions).<br>2 Fear of late effects of vaccination<br>3 Survived Covid-19 infection<br>4 Further or explanation:	Scale	-1, -8, -9	
AF08_04	Reason no vacc. - Resident: Further or explanation			Nominal		
AF09	Repeats. Quick tests	Does your institution use the offer for repetitive testing for employees, residents and visitors?	-9[a] not answered<br>1 Yes<br>2 No<br>3 Justification (optional):	Scale	-8, -9	
AF09_03	Repeated. Quick tests: Justification (optional)			Nominal		

Variable	Labeling	Question	Input	Measurement level	Missing values	Filter-variable
AF10	Measures first wave: fallback option (negative) or number of selected options.	What measures did you implement during the <u>first wave</u> of the pandemic?	-1 No specification	Scale		
AF10_01	Measures first wave: cohorting of individual living areas		1 not selected<br>2 selected	Nominal	-8	
AF10_02	Measures first wave: recording stop		1 not selected<br>2 selected	Nominal	-8	
AF10_03	Measures first wave: Establishment of a crisis team or crisis unit		1 not selected<br>2 selected	Nominal	-8	
AF10_04	Measures first wave: Measures to reduce contact (e.g., prohibition of visits by relatives, cessation of free...		1 not selected<br>2 selected	Nominal	-8	

AF10_05	Measures first wave: Expansion of existing hygiene processes	1 not selected<br>2 selected	Nominal	-8
AF10_06	Measures first wave: ban on residents leaving the building	1 not selected<br>2 selected	Nominal	-8
AF10_07	Measures first wave: Use of external advisory services (e.g., canton or infectious disease).	1 not selected<br>2 selected	Nominal	-8
AF10_08	Measures first wave: stop of activation offers	1 not selected<br>2 selected	Nominal	-8

AF10_09	Measures first wave: Preventive quarantine (in case of mild symptoms).		1 not selected<br>2 selected	Nominal	-8
AF10_10	Measures first wave: Further or explanation		1 not selected<br>2 selected	Nominal	-8
AF10_10a	Measures first wave: Further or explanation (open input)		1 not selected<br>2 selected	Nominal	
AF11	Measures first wave PWD	Have you implemented special measures for the care and support of people with dementia?	-9[a] not answered<br>-1[a] No specification<br>1 Yes, the following:<br>2 No	Scale	-1, -8, -9
AF11_01	Measures first wave PWD: Yes, the following.			Nominal	

Variable	Labeling	Question	Input	Measurement level	Missing values	Filter-variable
AF12	Measures current: fallback option (negative) or number of selected options	What measures are you <u>currently</u> implementing?	-1 No specification	Scale		
AF12_01	Current measures: Cohorting of individual living areas		1 not selected<br>2 selected	Nominal	-8	
AF12_02	Current measures: Recording stop		1 not selected<br>2 selected	Nominal	-8	
AF12_03	Current measures: Establishment of a crisis team or crisis unit s		1 not selected<br>2 selected	Nominal	-8	
AF12_04	Current measures: Measures to reduce contact (e.g., prohibition of visits by relatives, discontinuation of voluntary...		1 not selected<br>2 selected	Nominal	-8	

AF12_05	Current measures: Expansion of existing hygiene processes	1 not selected<br>2 selected	Nominal	-8
AF12_06	Current measures: ban on residents leaving the building	1 not selected<br>2 selected	Nominal	-8
AF12_07	Current measures: Use of external consulting services (e.g. canton or infectiology)	1 not selected<br>2 selected	Nominal	-8
AF12_08	Current measures: Stop of activation offers	1 not selected<br>2 selected	Nominal	-8

AF12_09	Current measures: Preventive quarantine (for mild symptoms)		1 not selected<br>2 selected	Nominal	-8
AF12_10	Current measures: Further or explanation		1 not selected<br>2 selected	Nominal	-8
AF12_10a	Current measures: Further or explanation (open entry)		1 not selected<br>2 selected	Nominal	
AF13	Measures current PWD	Are special measures currently being implemented for the care and support of people with dementia?	-9[a] not answered<br>-1[a] No specification<br>1 Yes, the following:<br>2 No	Scale	-1, -8, -9
AF13_01	Measures currently PWD: Yes, the following.			Nominal	

Variable	Labeling	Question	Input	Measurement level	Missing values	Filter-variable
AF14	Resources in first wave: fallback option (negative) or number of selected options.	What resources were NOT adequate during the <u>first wave</u> of the pandemic?	-1 No specification	Scale		
AF14_01	Resources in the first wave: Resources related to one's own expertise.		1 not selected<br>2 selected	Nominal	-8	
AF14_02	Resources in the first wave: resources related to the professional competence of the employees in nursing care		1 not selected<br>2 selected	Nominal	-8	
AF14_03	Resources in the first wave: Time resources		1 not selected<br>2 selected	Nominal	-8	

AF14_04	Resources in the first wave: resources of disinfection and hygiene materials.	1 not selected<br>2 selected	Nominal	-8
AF14_05	Resources in first wave: Resources on oxygen equipment	1 not selected<br>2 selected	Nominal	-8
AF14_06	Resources in the first wave: Resources of personal protective equipment	1 not selected<br>2 selected	Nominal	-8
AF14_07	Resources in first wave: Information resources on how to deal with the new situation.	1 not selected<br>2 selected	Nominal	-8

Variable	Labeling	Question	Input	Measurement level	Missing values	Filter-variable
AF14_08	Resources in first wave: Further or explanation		1 not selected<br>2 selected	Nominal	-8	
AF14_08a	Resources in first wave: Further or explanation (open input)		1 not selected<br>2 selected	Nominal		

Variable	***Labeling***	***Question***	***Input***	***Measurement level***	***Missing values***	***Filter-variable***
AF15	Resources current: fallback option (negative) or number of selected options.	What resources are <u>currently</u> NOT sufficiently available?	-1 No specification	Scale		

AF15_01	Resources up to date: Resources with reference to one's own expertise	1 not selected<br>2 selected	Nominal	-8
AF15_02	Resources up to date: Resources related to the professional competence of the nursing staff	1 not selected<br>2 selected	Nominal	-8
AF15_03	Current resources: Time resources	1 not selected<br>2 selected	Nominal	-8
AF15_04	Resources up to date: Resources of disinfection and hygiene materials	1 not selected<br>2 selected	Nominal	-8

AF15_05	Resources up to date: Oxygen equipment resources	1 not selected<br>2 selected	Nominal	-8
AF15_06	Resources up to date: Resources of personal protective equipment	1 not selected<br>2 selected	Nominal	-8
AF15_07	Resources up to date: information resources on how to deal with the new situation.	1 not selected<br>2 selected	Nominal	-8
AF15_08	Resources up to date: Further or explanation	1 not selected<br>2 selected	Nominal	-8

Variable	Labeling	Question	Input	Measurement level	Missing values	Filter-variable
AF15_08a	Resources current: Further or explanation (open input)		1 not selected<br>2 selected	Nominal		
AF16	Communication technology MA: fallback option (negative) or number of selected options.	What digital communication technology did you use during the pandemic to support residents in contact with relatives?	-1 No specification	Nominal		
AF16_01	Communication technology MA: Telephone		1 not selected<br>2 selected	Nominal	-8	
AF16_02	Communication technology MA: E-mail		1 not selected<br>2 selected	Nominal	-8	

Variable	Labeling	Question	Input	Measurement level	Missing values	Filter-variable
AF16_03	Communication technology MA: Messenger services		1 not selected<br>2 selected	Nominal	-8	
AF16_04	Communication technology MA: Video conferencing		1 not selected<br>2 selected	Nominal	-8	
AF16_05	Communication Technology MA: Telecare Technologies		1 not selected<br>2 selected	Nominal	-8	
AF16_06	Communication technology MA: Further		1 not selected<br>2 selected	Nominal	-8	
AF16_06a	Communication technology MA: Other (open input)		1 not selected<br>2 selected	Nominal		

Variable	***Labeling***	***Question***	***Input***	***Measurement level***	***Missing values***	***Filter-variable***
AF18	Team behavior change	Has teamwork changed with the pandemic? If so, how has collaboration changed?	-9[a] not answered<br>-1[a] No specification<br>1 More cohesion between team members<br>2 Less cohesion between team members<br>4 Further or explanation:	Scale	-1, -8, -9	

AF18_04	Team Behavior Change: Further or Explanation	Nominal

Variable	Labeling	Question	Input	Measurement level	Missing values	Filter-variable
AF19	Support - Institution 1st wave	During the <u>first wave</u>, how did you feel your institution supported you in dealing with the Corona situation?		Scale	-8, -9	
AF20	Support - Institution 2nd wave	During the <u>second wave</u>, how did you feel your institution supported you in dealing with the Corona situation?	-9[a] not answered<br>1 very good<br>2 rather good<br>3 rather poor<br>4 very poor<br>5 Not specified	Scale	-8, -9	
AF21	Support - Canton - Heiml. 1st wave	During the <u>first wave</u>, how did you feel supported by the leaders of your canton/municipality in dealing with the Corona situation?	-9[a] not answered<br>1 very good<br>2 rather good<br>3 rather poor<br>4 very poor<br>5 Not specified	Nominal	-8, -9	
AF22	Support - Canton - Heiml. - Heiml. 2nd wave	During the <u>second wave</u>, how did you feel supported by the leaders of your canton/municipality in dealing with the Corona situation?	-9[a] not answered<br>1 very good<br>2 rather good<br>3 rather poor<br>4 very poor<br>5 Not specified	Scale	-8, -9	

Variable	Labeling	Question	Input	Measurement level	Missing values	Filter-variable
AF23_01	Feelings MA - 1 wave : Fear	To what extent were the following feelings prevalent with you during the <u>first wave</u>?	-9[a] not answered<br>-1[a] No specification<br>1 not present<br>6 strongly present	Scale	-1, -8, -9	
AF23_02	Feelings MA - 1 wave : Anger		-9[a] not answered<br>-1[a] No specification<br>1 not present<br>6 strongly present	Scale	-1, -8, -9	
AF23_03	Feelings MA - 1 wave : Grief		-9[a] not answered<br>-1[a] No specification<br>1 not present<br>6 strongly present	Scale	-1, -8, -9	
AF23_04	Feelings MA - 1 wave : Confidence		-9[a] not answered<br>-1[a] No specification<br>1 not present<br>6 strongly present	Scale	-1, -8, -9	
AF23_05	Feelings MA - 1 wave : Feeling of being overwhelmed		-9[a] not answered<br>-1[a] No specification<br>1 not present<br>6 strongly present	Scale	-1, -8, -9	
AF23_06	Feelings MA - 1 wave : Sense of security		-9[a] not answered<br>-1[a] No specification<br>1 not present<br>6 strongly present	Scale	-1, -8, -9	
AF23_07	Feelings MA - 1 wave : Helplessness		-9[a] not answered<br>-1[a] No specification<br>1 not present<br>6 strongly present	Scale	-1, -8, -9	

AF23_08	Feelings MA - 1 wave :<br>Feeling challenged	-9[a] not answered<br>-1[a] No specification<br>1 not present<br>6 strongly present	Scale	-1, -8, -9
AF23_09	Feelings MA - 1 wave :<br>Exhaustion	-9[a] not answered<br>-1[a] No specification<br>1 not present<br>6 strongly present	Scale	-1, -8, -9
AF23_10	Feelings MA - 1 wave :<br>Insecurity	-9[a] not answered<br>-1[a] No specification<br>1 not present<br>6 strongly present	Scale	-1, -8, -9
AF23_11	Feelings MA - 1 wave :<br>Loneliness	-9[a] not answered<br>-1[a] No specification<br>1 not present<br>6 strongly present	Scale	-1, -8, -9

Variable	Labeling	Question	Input	Measurement level	Missing values	Filter-variable
AF24_01	Feelings MA - 2 wave : Fear	To what extent were the following feelings prevalent in you during the <u>second wave</u>?	-9[a] not answered<br>-1[a] No specification<br>1 not present<br>6 strongly present	Scale	-1, -8, -9	
AF24_02	Emotions MA - 2 Wave : Anger		-9[a] not answered<br>-1[a] No specification<br>1 not present<br>6 strongly present	Scale	-1, -8, -9	
AF24_03	Feelings MA - 2 wave : Grief		-9[a] not answered<br>-1[a] No specification<br>1 not present<br>6 strongly present	Scale	-1, -8, -9	
AF24_04	Feelings MA - 2 wave : Confidence		-9[a] not answered<br>-1[a] No specification<br>1 not present<br>6 strongly present	Scale	-1, -8, -9	
AF24_05	Feelings MA - 2 wave : Feeling of being overwhelmed		-9[a] not answered<br>-1[a] No specification<br>1 not present<br>6 strongly present	Scale	-1, -8, -9	
AF24_06	Feelings MA - 2 wave : Sense of security		-9[a] not answered<br>-1[a] No specification<br>1 not present<br>6 strongly present	Scale	-1, -8, -9	
AF24_07	Feelings MA - 2 wave : Helplessness		-9[a] not answered<br>-1[a] No specification<br>1 not present<br>6 strongly present	Scale	-1, -8, -9	
AF24_08	Feelings MA - 2 wave : Feeling challenged		-9[a] not answered<br>-1[a] No specification<br>1 not present<br>6 strongly present	Scale	-1, -8, -9	

AF24_09	Feelings MA - 2 wave :<br>Exhaustion	-9[a] not answered<br>-1[a] No specification<br>1 not present<br>6 strongly present	Scale	-1, -8, -9
AF24_10	Feelings MA - 2 wave :<br>Insecurity	-9[a] not answered<br>-1[a] No specification<br>1 not present<br>6 strongly present	Scale	-1, -8, -9
AF24_11	Feelings MA - 2 Wave :<br>Loneliness	-9[a] not answered<br>-1[a] No specification<br>1 not present<br>6 strongly present	Scale	-1, -8, -9

Variable	Labeling	Question	Input	Measurement level	Missing values	Filter-variable
AF25_01	Feelings MA - Current: Fear	To what extent are the following feelings <u>currently</u> prevalent with you?	-9[a] not answered<br>-1[a] No specification<br>1 not present<br>6 strongly present	Scale	-1, -8, -9	
AF25_02	Feelings MA - Current: Anger		-9[a] not answered<br>-1[a] No specification<br>1 not present<br>6 strongly present	Scale	-1, -8, -9	
AF25_03	Feelings MA - Current: Grief		-9[a] not answered<br>-1[a] No specification<br>1 not present<br>6 strongly present	Scale	-1, -8, -9	
AF25_04	Feelings MA - Current: Confidence		-9[a] not answered<br>-1[a] No specification<br>1 not present<br>6 strongly present	Scale	-1, -8, -9	
AF25_05	Feelings MA - Current: Feeling overwhelmed		-9[a] not answered<br>-1[a] No specification<br>1 not present<br>6 strongly present	Scale	-1, -8, -9	
AF25_06	Feelings MA - Current: Sense of security		-9[a] not answered<br>-1[a] No specification<br>1 not present<br>6 strongly present	Scale	-1, -8, -9	
AF25_07	Feelings MA - Current: Helplessness		-9[a] not answered<br>-1[a] No specification<br>1 not present<br>6 strongly present	Scale	-1, -8, -9	
AF25_08	Feelings MA - Current: Feeling challenged		-9[a] not answered<br>-1[a] No specification<br>1 not present<br>6 strongly present	Scale	-1, -8, -9	

AF25_09	Feelings MA - Current: Exhaustion		-9[a] not answered<br>-1[a] No specification<br>1 not present<br>6 strongly present	Scale	-1, -8, -9	
AF25_10	Feelings MA - Current: Uncertainty		-9[a] not answered<br>-1[a] No specification<br>1 not present<br>6 strongly present	Scale	-1, -8, -9	
AF25_11	Feelings MA - Current: Loneliness		-9[a] not answered<br>-1[a] No specification<br>1 not present<br>6 strongly present	Scale	-1, -8, -9	

Variable	***Labeling***	***Question***	***Input***	***Measurement level***	***Missing values***	***Filter-variable***
AF26_01	Feelings BW - 1 wave: Fear	To what extent were the following feelings prevalent among residents during the <u>first wave</u>?	-9[a] not answered<br>-1[a] No specification<br>1 not present<br>6 strongly present	Scale	-1, -8, -9	
AF26_02	Feelings BW - 1 wave: Anger		-9[a] not answered<br>-1[a] No specification<br>1 not present<br>6 strongly present	Scale	-1, -8, -9	
AF26_03	Feelings BW - 1 wave: Grief		-9[a] not answered<br>-1[a] No specification<br>1 not present<br>6 strongly present	Scale	-1, -8, -9	
AF26_04	Feelings BW - 1 wave: Confidence		-9[a] not answered<br>-1[a] No specification<br>1 not present<br>6 strongly present	Scale	-1, -8, -9	
AF26_05	Feelings BW - 1 wave: Feeling of being overwhelmed		-9[a] not answered<br>-1[a] No specification<br>1 not present<br>6 strongly present	Scale	-1, -8, -9	

AF26_06	Feelings BW - 1 wave:<br>Sense of security	-9[a] not answered<br>-1[a] No specification<br>1 not present<br>6 strongly present	Scale	-1, -8, -9
AF26_07	Feelings BW - 1 wave:<br>Helplessness	-9[a] not answered<br>-1[a] No specification<br>1 not present<br>6 strongly present	Scale	-1, -8, -9
AF26_08	Feelings BW - 1 wave:<br>Feeling challenged	-9[a] not answered<br>-1[a] No specification<br>1 not present<br>6 strongly present	Scale	-1, -8, -9
AF26_09	Feelings BW - 1 wave:<br>exhaustion	-9[a] not answered<br>-1[a] No specification<br>1 not present<br>6 strongly present	Scale	-1, -8, -9
AF26_10	Feelings BW - 1 wave:<br>Insecurity	-9[a] not answered<br>-1[a] No specification<br>1 not present<br>6 strongly present	Scale	-1, -8, -9
AF26_11	Feelings BW - 1 wave:<br>Loneliness	-9[a] not answered<br>-1[a] No specification<br>1 not present<br>6 strongly present	Scale	-1, -8, -9

Variable	Labeling	Question	Input	Measurement level	Missing values	Filter-variable
AF27_01	Feelings BW - 2 Wave: Fear	To what extent were the following feelings prevalent among residents during the <u>second wave</u>?	-9[a] not answered<br>-1[a] No specification<br>1 not present<br>6 strongly present	Scale	-1, -8, -9	
AF27_02	Feelings BW - 2 wave: Anger		-9[a] not answered<br>-1[a] No specification<br>1 not present<br>6 strongly present	Scale	-1, -8, -9	
AF27_03	Feelings BW - 2 wave: Grief		-9[a] not answered<br>-1[a] No specification<br>1 not present<br>6 strongly present	Scale	-1, -8, -9	
AF27_04	Feelings BW - 2 Wave: Confidence		-9[a] not answered<br>-1[a] No specification<br>1 not present<br>6 strongly present	Scale	-1, -8, -9	
AF27_05	Feelings BW - 2 wave: Feeling of being overwhelmed		-9[a] not answered<br>-1[a] No specification<br>1 not present<br>6 strongly present	Scale	-1, -8, -9	
AF27_06	Feelings BW - 2 wave: Sense of security		-9[a] not answered<br>-1[a] No specification<br>1 not present<br>6 strongly present	Scale	-1, -8, -9	
AF27_07	Feelings BW - 2 wave: Helplessness		-9[a] not answered<br>-1[a] No specification<br>1 not present<br>6 strongly present	Scale	-1, -8, -9	
AF27_08	Feelings BW - 2 wave: Feeling challenged		-9[a] not answered<br>-1[a] No specification<br>1 not present<br>6 strongly present	Scale	-1, -8, -9	

AF27_09	Feelings BW - 2 Wave: Exhaustion	-9[a] not answered<br>-1[a] No specification<br>1 not present<br>6 strongly present	Scale	-1, -8, -9
AF27_10	Feelings BW - 2 Wave: Insecurity	-9[a] not answered<br>-1[a] No specification<br>1 not present<br>6 strongly present	Scale	-1, -8, -9
AF27_11	Feelings BW - 2 Wave: Loneliness	-9[a] not answered<br>-1[a] No specification<br>1 not present<br>6 strongly present	Scale	-1, -8, -9

Variable	Labeling	Question	Input	Measurement level	Missing values	Filter-variable
AF28_01	Feelings BW - Current: Fear	To what extent are the following feelings <u>currently</u> prevalent among residents?	-9[a] not answered<br>-1[a] No specification<br>1 not present<br>6 strongly present	Scale	-1, -8, -9	
AF28_02	Feelings BW - Current: Anger		-9[a] not answered<br>-1[a] No specification<br>1 not present<br>6 strongly present	Scale	-1, -8, -9	
AF28_03	Feelings BW - Current: Grief		-9[a] not answered<br>-1[a] No specification<br>1 not present<br>6 strongly present	Scale	-1, -8, -9	
AF28_04	Feelings BW - Current: Confidence		-9[a] not answered<br>-1[a] No specification<br>1 not present<br>6 strongly present	Scale	-1, -8, -9	
AF28_05	Feelings BW - Current: Feeling of being overwhelmed		-9[a] not answered<br>-1[a] No specification<br>1 not present<br>6 strongly present	Scale	-1, -8, -9	
AF28_06	Feelings BW - Current: Sense of security		-9[a] not answered<br>-1[a] No specification<br>1 not present<br>6 strongly present	Scale	-1, -8, -9	
AF28_07	Feelings BW - Current: Helplessness		-9[a] not answered<br>-1[a] No specification<br>1 not present<br>6 strongly present	Scale	-1, -8, -9	
AF28_08	Feelings BW - Current: Feeling challenged		-9[a] not answered<br>-1[a] No specification<br>1 not present<br>6 strongly present	Scale	-1, -8, -9	

AF28_09	Feelings BW - Current: Exhaustion	-9[a] not answered<br>-1[a] No specification<br>1 not present<br>6 strongly present	Scale	-1, -8, -9
AF28_10	Feelings BW - Current: Uncertainty	-9[a] not answered<br>-1[a] No specification<br>1 not present<br>6 strongly present	Scale	-1, -8, -9
AF28_11	Feelings BW - Current: Loneliness	-9[a] not answered<br>-1[a] No specification<br>1 not present<br>6 strongly present	Scale	-1, -8, -9

Variable	*Labeling*	*Question*	*Input*	*Measurement level*	*Missing values*	*Filter-variable*
AF34	Perception Inhab. Alg	Have you noticed new behaviors in residents WITHOUT dementia?	-9[a] not answered<br>-1[a] No specification<br>6 Yes, the following:<br>7 No	Scale	-1, -8, -9	
AF34_06	Perception Inhab. Alg: Yes, following			Nominal		
AF29	Perception of PWD	Have you noticed any new behaviors in residents WITH dementia?	-9[a] not answered<br>-1[a] No specification<br>6 Yes, the following:<br>7 No	Scale	-1, -8, -9	
AF29_06	Perception of PWD: Yes, the following			Nominal		
AF30	Special measures for PWD	Are there any other aspects you would like to share with us regarding the topic of "COVID-19 and dementia"?	-9[a] not answered<br>-1[a] No specification<br>6 Yes, the following:<br>7 No	Scale	-1, -8, -9	
AF30_06	Special measures for PWD: Yes, the following			Nominal		

Variable	Labeling	Question	Input	Measurement level	Missing values	Filter-variable
AF31	Appreciation: fallback option (negative) or number of selected options.	From which of these groups do you feel your work performance is <u>NOT</u> adequately valued?	-1 No specification	Scale		
AF31_01	Appreciation: Politics national		1 not selected<br>2 selected	Nominal	-8	
AF31_02	Appreciation: policy cantonal		1 not selected<br>2 selected	Nominal	-8	
AF31_03	Appreciation: health care system		1 not selected<br>2 selected	Nominal	-8	
AF31_04	Appreciation: Population		1 not selected<br>2 selected	Nominal	-8	
AF31_05	Appreciation: Residents		1 not selected<br>2 selected	Nominal	-8	
AF31_06	Appreciation: relatives		1 not selected<br>2 selected	Nominal	-8	

AF31_07	Appreciation: Further or explanation	1 not selected<br>2 selected	Nominal	-8
AF31_07a	Appreciation: Further or explanation (open input)		Nominal	

Variable	Labeling	Question	Input	Measurement level	Missing values	Filter-variable
AF32	Wishes/Requests: Fallback option (negative) or number of options selected.	What wishes or demands do you have for politicians, the population and other groups, with regard to your work in care during the Corona pandemic?		Scale		
AF32_01	Wishes/Requests: [No description] 01			Nominal	-8	
AF32_01a	Wishes/Requests: [No description] 01 (open input)			Nominal		
AF32_02	Wishes/Requests: [No description] 02			Nominal	-8	
AF32_02a	Wishes/Requests: [No description] 02 (open input)			Nominal		

AF32_03	Wishes/Requests: [No description] 03	Nominal	-8
AF32_03a	Wishes/Requests: [No description] 03 (open input)	Nominal	

Variable	Labeling	Question	Input	Measurement level	Missing values	Filter-variable
OF02_01	Free text AF19: Reasons			Nominal		
OF03_01	Free text AF20: Reasons			Nominal		
OF04_01	Free text AF21: Reasons			Nominal		
OF05_01	Free text AF22: Reasons			Nominal		
OF06_01	Free text AF23: Further or explanation			Nominal		
OF07_01	Free text AF24: Further or explanation			Nominal		
OF08_01	Free text AF25: Further or explanation			Nominal		
OF09_01	Free text AF26: Further or explanation			Nominal		
OF10_01	Free text AF27: Further or explanation			Nominal		

OF11_01	Free text AF28: Further or explanation	Nominal

Variable	Labeling	Question	Input	Measurement level	Missing values	Filter-variable
TIME001	Dwell time page 1			Scale		
TIME002	Dwell time page 2			Scale		
TIME003	Dwell time Page 3			Scale		
TIME004	Dwell time Page 4			Scale		
TIME005	Dwell time Page 5			Nominal		
TIME006	Dwell time Page 6			Nominal		
TIME007	Dwell time Page 7			Scale		
TIME008	Dwell time Page 8			Scale		
TIME009	Dwell time Page 9			Scale		
TIME010	Dwell time Page 10			Scale		
TIME011	Dwell time Page 11			Scale		
TIME012	Dwell time Page 12			Scale		
TIME013	Dwell time Page 13			Scale		

TIME014	Dwell time Page 14	Scale
TIME015	Dwell time Page 15	Scale
TIME_SUM	Total length of stay (without outliers)	Scale
MAILSENT	Sending time of the invitation mail (only for non-anonymous addressees)	Scale
LASTDATA	Time when the record was last changed	Scale
FINISHED	Was the survey completed (last page reached)?	Nominal
Q_VIEWER	Did the participant only look at the questionnaire without answering the mandatory questions?	Nominal
LASTPAGE	Page that the participant last edited	Scale

MAXPAGE	Last page edited in the questionnaire	Scale
MISSING	Percentage of missing answers	Nominal
MISSREL	Proportion of missing responses (weighted by relevance)	Nominal
TIME_RSI	Malus points for fast filling	Scale
DEG_TIME	Malus points for fast filling	Scale
